# Psychobiotics and Trace Elements in Neurodegenerative Diseases: From Independent Modulators to Potential Interactions Within the Microbiota–Gut–Brain Axis—Narrative Review

**DOI:** 10.3390/ijms27219955

**Published:** 2026-11-06

**Authors:** Weronika Sokólska, Karolina Orywal

**Affiliations:** Department of Biochemical Diagnostics, Medical University of Bialystok, Waszyngtona 15A, 15-269 Bialystok, Poland

**Keywords:** neurodegenerative disease, microbiota–gut–brain axis, psychobiotics, trace elements, neuroinflammation

## Abstract

The microbiota–gut–brain axis represents an important bidirectional communication network linking the gastrointestinal tract and the central nervous system and may contribute to processes involved in neurodegeneration. Increasing attention has been directed toward psychobiotics as potential modulators of the microbiota–gut–brain axis, while accumulating evidence also indicates that disturbed trace-element homeostasis may contribute to the pathogenesis of neurodegenerative diseases. This review summarizes current evidence on the roles of psychobiotics and trace elements and examines their potential interplay within the microbiota–gut–brain axis. The review discusses bidirectional interactions between the gut microbiota and trace elements, the potential effects of psychobiotic interventions on trace-element homeostasis, and the possible implications of these interactions for neurodegenerative processes. Taken together, current findings indicate that the gut microbiota, psychobiotics, and trace elements may constitute an interconnected system influencing intestinal and systemic homeostasis as well as biological processes relevant to nervous system function. However, direct evidence demonstrating the clinical relevance of interactions between these components remains limited. Moreover, available evidence is largely based on experimental studies, while clinical findings remain scarce and heterogeneous. Further characterization of this interplay may improve our understanding of mechanisms underlying neurodegeneration and provide a basis for the development of more targeted and personalized approaches to supporting neurological health.

## 1. Introduction

Neurodegenerative diseases comprise a heterogeneous group of disorders characterized by diverse pathological mechanisms, including the accumulation of misfolded proteins, neuroinflammation, oxidative stress, and impaired neuronal signaling. The most prevalent neurodegenerative diseases include Alzheimer’s disease (AD), Parkinson’s disease (PD), amyotrophic lateral sclerosis (ALS), and multiple sclerosis (MS) [1,2,3]. As life expectancy has increased in many countries over recent decades, demographic transitions have resulted in a growing proportion of older individuals, accompanied by a rising prevalence of neurodegenerative diseases [4].

Alzheimer’s disease accounts for approximately 60–70% of all cases of dementia. Between 1992 and 2021, the global number of AD cases increased from 4.078 million to 9.837 million [5]. Parkinson’s disease, the second most common neurodegenerative disease worldwide, has similarly shown a substantial increase in prevalence, rising by 274% from 3.15 million to 11.77 million cases [6,7]. Despite substantial advances in understanding the molecular mechanisms underlying neurodegeneration, currently available treatments remain predominantly symptomatic and are unable to effectively halt or reverse disease progression. Combined with the increasing disease burden associated with population aging, this makes neurodegenerative diseases one of the major challenges facing modern medicine and public health. Consequently, increasing attention is being devoted to identifying novel pathogenic mechanisms and potential therapeutic targets [8].

In recent years, the microbiota–gut–brain axis (MGBA) has attracted considerable scientific interest. The MGBA is a bidirectional communication system involving neuronal, immune, endocrine, and metabolic pathways that enables reciprocal interactions between the gut microbiota and the central nervous system [9]. Dysregulation of this complex network may play an important role in the initiation and progression of neurodegenerative processes [2,3]. Preclinical evidence also indicates a relationship between metal exposure, brain metal homeostasis, and gut microbial composition. In rats, aluminum exposure was associated with reduced brain levels of zinc, iron, and manganese, accompanied by changes in the gut bacterial community [10]. Large-scale reference mapping has further highlighted the extensive diversity of the human gut microbiome, including hundreds of previously uncharacterized species [11]. More broadly, research has increasingly addressed microbiota-targeted interventions and the relationship between trace element availability and intestinal microbial function, although these research areas have largely been investigated independently [12,13,14,15].

Increasing evidence, however, suggests that psychobiotics and trace elements may modulate overlapping biological pathways, affecting, among other processes, gut microbiota composition, intestinal barrier integrity, immune responses, oxidative stress, and mitochondrial function [12,14,15]. Moreover, gut microorganisms and trace elements may interact bidirectionally, with trace-element availability and chemical form potentially influencing microbial growth and metabolic activity, while gut microorganisms may affect the bioavailability and metabolism of selected elements. Understanding these interrelationships may open new avenues for the development of more comprehensive and personalized therapeutic strategies for neurodegenerative diseases. Previous reviews have addressed related but distinct aspects of this field. Mörkl et al. examined probiotics and the MGBA, with a particular focus on psychiatry [16]. Forero-Rodríguez et al. explored the metal–microbiome–gut–brain axis in Parkinson’s disease from a systems toxicology perspective [17], whereas Tinkov et al. focused on the potential contribution of the gut microbiota to manganese-induced neurotoxicity [18]. More recently, Cao et al. discussed the aging gut–brain axis in relation to dietary polyphenols and metal exposure [19]. These reviews provide important background for understanding the relationships between gut microbiota, metal homeostasis, and brain function, but focus on particular components, disorders, or exposures.

The present review builds on this literature by integrating evidence on psychobiotics and selected trace elements as independent and potentially interacting modulators of neurodegenerative processes. Unlike previous reviews focusing primarily on individual components of the microbiota–gut–brain axis, specific metals, or particular neurodegenerative conditions, this review brings together evidence from these two research areas to examine their potential interactions and shared mechanistic pathways. Particular attention is given to their effects on gut–brain signaling, neuroinflammation, oxidative stress, ferroptosis, and metal homeostasis, while distinguishing direct evidence from combined psychobiotic–trace element interventions from indirect evidence based on separate studies or overlapping mechanisms. In addition, the review critically evaluates the limitations of the available evidence and identifies priorities for future research.

In this review, we summarize the current evidence regarding psychobiotics and trace elements as interconnected modulators of the microbiota–gut–brain axis in neurodegenerative diseases. Particular attention is given to the mechanisms through which these two approaches may act synergistically on processes associated with neurodegeneration, as well as to the limitations of the available evidence and the directions for future research required to develop more effective therapeutic strategies. The available evidence is considered in terms of its mechanistic, preclinical, and clinical relevance. Although neurodegenerative disorders constitute a diverse group of diseases, this review focuses primarily on Alzheimer’s disease and Parkinson’s disease, as the most data on gut microbiota, trace element homeostasis, and psychobiotic interventions are available for these conditions.

## 2. The Gut–Brain Axis

### 2.1. Structure and Components of the Microbiota–Gut–Brain Axis

**Figure 1:**
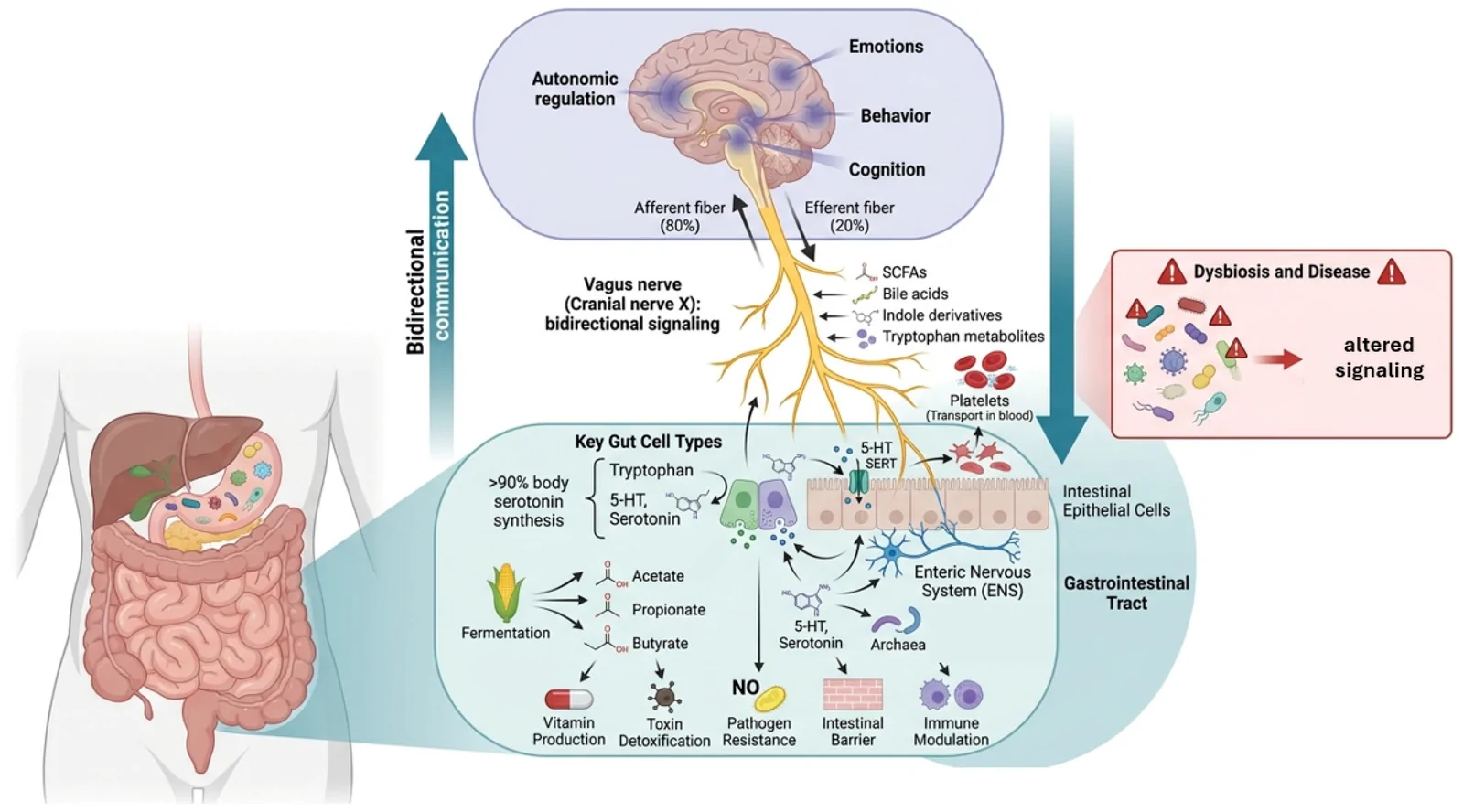
The microbiota–gut–brain axis.

The microbiota–gut–brain axis (MGBA) has emerged as a highly intriguing and clinically relevant area of research in recent years. Increasing efforts have been directed toward understanding the interactions between two seemingly distinct and autonomous systems: the gastrointestinal tract and the central and peripheral nervous systems. However, accumulating evidence from intensive research over the past years indicates the existence of numerous interdependencies and shared biological pathways connecting these systems, largely mediated by microorganisms inhabiting the gastrointestinal tract (Figure 1) [16].

The MGBA refers to a complex communication network involving interactions among its individual components. The human gut is colonized by diverse microbial communities comprising bacteria, fungi, viruses, archaea, and eukaryotes, collectively referred to as the gut microbiota [20]. The human gut microbiota is established early in life and undergoes dynamic changes during the first years of life, while its composition and function may continue to vary in response to host-related and environmental factors [20,21]. Importantly, gut microbiota composition and activity can also be modulated by dietary interventions. In an MPTP-induced mouse model of Parkinson’s disease, a four-week isoflavone-enriched diet containing genistein (0.24 g/kg diet) and daidzein (0.22 g/kg diet) promoted Lactobacillus-associated serotonin production and attenuated motor deficits and dopaminergic neuronal loss [22].

The gut microbiota performs a wide range of essential physiological functions. These include the fermentation of otherwise indigestible dietary components into absorbable metabolites, synthesis of essential vitamins, detoxification and elimination of potentially harmful compounds, resistance to pathogen colonization, maintenance and reinforcement of intestinal barrier integrity, and stimulation and regulation of immune system activity [23].

### 2.2. Communication Mechanisms

The bidirectional interaction between the gut microbiota and the host is enabled by an interconnected network of signaling pathways, which can be divided into several main categories. One of the best-characterized mechanisms of communication involves metabolic interactions. As a result of the fermentation of indigestible dietary components by gut bacteria, biologically active metabolites are produced that exert both local and systemic effects [24]. Gut microbiota-dependent signaling also contributes to neuroimmune homeostasis. Experimental studies in germ-free mice have demonstrated that microbiota is required for normal microglial maturation and function, with short-chain fatty acids contributing to the regulation of microglial homeostasis [25]. Short-chain fatty acids (SCFAs) are the end products of the fermentation of indigestible dietary fiber by bacteria inhabiting the colon. The main SCFAs include acetate, propionate, and butyrate. They can cross the intestinal mucosa and enter the systemic circulation.

Psychobiotic interventions and dietary strategies, including fermentable fiber intake, are being explored as complementary approaches to modulating gut microbial activity and gut–brain signaling in neurodegenerative disorders, although evidence for synergistic effects remains limited [26]. Fermentable dietary fibers provide substrates for SCFA-producing bacteria. In mice, dietary supplementation with 2.5% inulin for two weeks increased acetate, butyrate, and propionate concentrations in the cecum, as well as butyrate and propionate levels in portal plasma. In a separate eight-week experiment, microglia isolated from inulin-fed mice exhibited reduced TNF-α secretion following in vitro LPS stimulation. Consistently, acetate and butyrate (100 μM) reduced LPS-induced inflammatory cytokine expression and NF-κB activation in primary mouse microglia [27]. Dietary fiber may also influence microbial tryptophan metabolism. In a study combining a defined three-species microbial community, human fecal cultures, and fecal microbiota-transplanted gnotobiotic mice, fiber-dependent metabolic interactions reduced indole production and favored the formation of beneficial tryptophan-derived metabolites, particularly indolelactic acid (ILA) [28]. These metabolic effects may intersect with intestinal barrier function and neuroimmune signaling. Dysbiosis-associated impairment of intestinal barrier integrity may facilitate the translocation of bacterial components, including LPS, promoting systemic inflammation and potentially affecting blood–brain barrier integrity and microglial activity [29,30]. Together, these findings suggest that psychobiotic and dietary interventions may influence interconnected metabolic and neuroinflammatory pathways relevant to neurodegeneration, although their combined effects require further investigation. Enterochromaffin (EC) cells of the intestine, which are the most abundant type of neuroendocrine cells in the gastrointestinal tract, participate in signal processing [31].

Although serotonin (5-hydroxytryptamine (5-HT)) is widely recognized as a neurotransmitter in the central nervous system, most serotonin in the body is produced peripherally, with enterochromaffin cells in the gastrointestinal tract being its primary source (>90%). SCFAs, which are microbial metabolites, play an important role in promoting 5-HT biosynthesis [32]. This compound is synthesized from tryptophan in the presence of the rate-limiting enzyme tryptophan hydroxylase (Tph/TPH) 1 [33]. To a much lesser extent, its production in the myenteric plexus is mediated by the second isoform, TPH2 [32,34]. After release, serotonin acts locally on enteric and sensory pathways. Its extracellular availability is regulated in part by SERT-mediated uptake by intestinal epithelial cells, while circulating serotonin is largely taken up by platelets [33].

Following its release from enterochromaffin cells, serotonin can enter the circulation, where it is taken up by platelets via the serotonin transporter (SERT). Gut-derived serotonin can reach the liver through the portal circulation and may also contribute to signaling in peripheral target tissues [35]. In response to chemical and mechanical stimuli within the intestinal lumen, released 5-HT regulates gastrointestinal motility, stimulates secretory responses, and mediates visceral sensory signaling through interactions with the enteric nervous system (ENS) and sensory neurons [36]. Research suggests that intestinal serotonergic signaling may be associated with gut microbiota. In cell-based experiments, Yano et al. demonstrated that indigenous spore-forming bacteria stimulate intestinal serotonin biosynthesis and thereby influence gastrointestinal and systemic serotonergic signaling [34]. Separately, experiments in PC12 cells demonstrated that propionate and butyrate can modulate gene expression, including CREB-dependent catecholaminergic neurotransmission [37]. Nutrient-induced serotonin secretion also varies across gastrointestinal regions [38]. In turn, the findings of Moretti et al. indicate that specific members of the human gut microbiome are capable of directly producing bioactive serotonin (in vitro). The researchers suggest that colonization with these microorganisms affects serotonergic signaling in the colon, intestinal innervation, and intestinal transit [39].

The influence of microbiota-derived signals on intestinal serotonergic signaling is not limited to local gastrointestinal functions. Changes in the intestinal environment must also be conveyed to the central nervous system, which requires communication pathways connecting the gut and the brain. One of the key neuronal pathways involved in this bidirectional communication is the vagus nerve. It is the tenth cranial nerve and consists of afferent fibers (80%) and efferent fibers (20%) [40]. As the main component of the parasympathetic division of the autonomic nervous system, it participates in the regulation of visceral functions, while its afferent fibers transmit information from the gastrointestinal tract to structures within the brainstem [41]. Information received by vagal afferent fibers can be modulated by the composition and metabolic activity of the gut microbiota. Gut microorganisms and their metabolites, such as SCFAs and indole derivatives, as well as host-derived metabolites modified by the gut microbiota-including bile acid derivatives and tryptophan metabolites-can interact with epithelial cells, enteroendocrine cells, and components of the enteric nervous system, thereby influencing the activity of vagal afferent fibers [42,43]. In this way, signals generated by the microbiota can be converted into neuronal impulses and transmitted to the central nervous system [44]. The vagus nerve represents an important pathway of communication between the gastrointestinal tract and the CNS. In mice, FFAR3 expression in vagal neurons has been implicated in the regulation of feeding behavior and the anorectic response to propionate [45]. These findings suggest that microbiota-derived metabolites may contribute to gut–brain communication, including pathways involved in energy balance. Although the potential implications of this mechanism for cognition and neurodegeneration remain to be explored, it provides an example of how microbial metabolites may influence host physiology.

### 2.3. Dysbiosis

Intestinal dysbiosis is a phenomenon characterized by changes in the composition, diversity, or functionality of the microbial communities inhabiting the gastrointestinal tract, which consequently disrupts their physiological interaction with the host. Given that there is no single universal model of microbiota, dysbiosis should not be defined solely as the loss of specific microorganisms or an increase in the abundance of potentially pathogenic taxa, but also as a disruption of the microbiota’s functional potential. Before focusing on the consequences of dysbiosis, it is important to emphasize that microorganisms colonizing the gastrointestinal tract perform several important functions in the body, serving as a metabolically and immunologically active component of the organism. Furthermore, the microbiota serves as a crucial component of the barrier against colonization of the gastrointestinal tract by potentially pathogenic microorganisms, including through competition for nutrients and adhesion sites, as well as by stimulating the host to produce antimicrobial compounds. Bacteria of the genus *Bifidobacterium*, which are an important component of the gut microbiota, could synthesize certain vitamins de novo, particularly B vitamins and vitamin K. These compounds can support both the metabolism of microorganisms and the host’s vitamin homeostasis [46,47]. The gut microbiota is also a key regulator of mucosal immune homeostasis. Microbial components and metabolites interact with epithelial and immune system cells, modulating cytokine production, the differentiation of immune cells, and the balance between inflammatory and regulatory responses [48].

Dysbiosis results in a reduction in key functions of the microbiota. This leads to decreased production of beneficial metabolites, particularly butyrate, which can result in impaired epithelial cell function, changes in the expression of tight junction proteins, and compromised intestinal barrier integrity. Increased intestinal permeability may facilitate the translocation of microbiota-derived components, including lipopolysaccharide (LPS), into circulation. LPS, as a component of the outer membrane of Gram-negative bacteria, can activate Toll-like receptor 4 (TLR4) present on immune system cells, initiating a signaling cascade involving, among others, the MyD88/NF-κB pathway and leading to increased production of pro-inflammatory cytokines such as TNF-α, IL-1β, and IL-6. In this way, disruption of the intestinal barrier may contribute to chronic activation of the immune system and the development of a systemic pro-inflammatory environment [30,49,50,51].

The systemic consequences of dysbiosis may also affect the functioning of the central nervous system. Circulating pro-inflammatory microbial metabolites and cytokines may affect the integrity of the blood–brain barrier (BBB) and the activity of endothelial and neuroimmune cells. This may lead to the activation of microglia and the development of local neuroinflammation, which, under certain conditions, contributes to damage to dopaminergic neurons in the substantia nigra (SN). By impairing neuronal function and viability, this process contributes to the development of cognitive and motor disorders [52,53,54,55]. The described relationships indicate that dysbiosis may affect the functioning of the nervous system through interrelated metabolic, immunological, and neuronal mechanisms, serving as a potential link between disturbances in intestinal homeostasis and neuroinflammation and neurodegenerative processes. A growing body of experimental and clinical data points to changes in the composition and metabolic activity of the microbiota in patients with neurodegenerative diseases, including Parkinson’s disease and Alzheimer’s disease [56,57,58,59]. At the same time, the nature of this relationship remains complex, and dysbiosis may, on the one hand, be a factor promoting the development of neurodegenerative processes and, on the other hand, a consequence of them. For this reason, strategies aimed at modulating the gut microbiota and restoring its beneficial metabolic and immunological functions take on particular significance. One promising avenue is psychobiotics, whose potential effects extend beyond modifying the composition of the microbiota to include influencing the communication pathways of the microbiota–gut–brain axis.

## 3. Psychobiotics as Modulators of Neurodegeneration

The concept of psychobiotics arose from a growing awareness that modulation of the gut microbiota can influence brain function via the microbiota–gut–brain axis. Initially, this term referred primarily to probiotic microorganisms that can benefit mental health through interactions with gut microbiota. Currently, its meaning has expanded to include microbiota-targeted interventions that can modulate gut–brain signaling (GBA) [60,61,62].

Among the bacterial genera commonly investigated for probiotic effects and their potential relevance to psychobiotic mechanisms are *Lactobacillus* and *Bifidobacterium*. Evidence from experimental models suggests that selected probiotic strains may influence neuroactive signaling and inflammatory responses, although these effects are strain- and model-dependent. For example, Cuevas-Carbonell et al. investigated an orally administered probiotic mixture containing *Lacticaseibacillus rhamnosus* GG and *Bifidobacterium animalis* subsp. *lactis* BB-12 in male Wistar rats with 6-hydroxydopamine (6-OHDA)-induced hemiparkinsonism. The mixture was administered for two weeks before and three weeks after unilateral injection of 6-OHDA into the right striatum. Probiotic treatment attenuated selected motor deficits, striatal microglial activation, and dopaminergic neuronal loss [63]. The effects of *Bifidobacterium longum* CCFM1029 on tryptophan metabolism were investigated in three complementary models [64]. In vitro, a resting-cell culture of *B. longum* CCFM1029 at 8.5 × 10^8^ CFU/mL was used to assess the production of tryptophan-derived metabolites, including indole-3-carbaldehyde (I3C). In a separate experiment, six-week-old female C57BL/6 mice with DNFB-induced atopic dermatitis-like symptoms received *B. longum* CCFM1029 by daily oral gavage at 10^9^ CFU in 0.2 mL, beginning one week before disease induction and continuing throughout the experiment. In the clinical component, patients with atopic dermatitis received 10^9^ CFU of *B. longum* CCFM1029 daily for eight weeks in a randomized, placebo-controlled intervention. The study reported changes in tryptophan-related metabolites and immune-associated outcomes across the experimental and clinical models [64]. Although this study did not directly investigate psychobiotic effects or neurodegeneration, it provides complementary evidence that probiotic-associated metabolic changes may influence host signaling. Furthermore, Sule et al. investigated the activity of 3,4-dihydroxymandelic acid (DHMA), a norepinephrine metabolite, using in vitro experiments with enterohemorrhagic *Escherichia coli* O157 strain 86-24. For the assessment of virulence-gene expression, bacterial cultures were exposed to 50 μM DHMA; chemotactic responses were evaluated using DHMA concentration gradients [65]. The investigators also analyzed fecal samples from conventional specific-pathogen-free C57BL/6 mice aged 4–12 weeks and germ-free mice aged 3–8 weeks. DHMA was detected in the fecal samples of conventional mice but not in those of germ-free animals, supporting a contribution of the commensal microbiota to its production [65]. This finding illustrates the broader biological activity of microbiota-derived metabolites but does not constitute direct evidence of psychobiotic activity or neuroprotective effects.

The exact mechanisms of action of psychobiotics are not yet fully understood; however, research suggests several potential pathways through which they may function. One of these is their effect on the hypothalamic–pituitary–adrenal (HPA) axis. The HPA axis is responsible for the body’s responses to physiological and physical stress and for maintaining homeostasis [66]. Chronic stress can dysregulate the HPA axis, alter glucocorticoid signaling, and contribute to increased intestinal permeability and changes in gut microbiota composition [67]. Furthermore, stressors—both physiological and psychological—can cause tight junctions (TJs) in the gastrointestinal tract to become leaky. Consequently, this leads to the unintended release of LPS-containing gut bacteria, further amplifying the initial stress response. Experimental studies suggest that modulation of the gut microbiota by probiotic microorganisms may influence HPA axis activity through interrelated immunological, metabolic, and neuronal mechanisms [68,69]. For example, it has been shown that administering probiotics to mice attenuated LPS-induced changes in the expression of glucocorticoid receptors in the paraventricular nucleus of the hypothalamus [68]. In contrast, rats subjected to maternal separation stress exhibited hypercorticosteronemia, increased intestinal permeability, and gut microbiome dysbiosis; however, treatment with *Bifidobacterium bifidum* G9-1 prevented this phenomenon [70]. These findings suggest that gut microbiota may potentially influence the central mechanisms involved in the regulation of the HPA axis. Clinical studies have further demonstrated the effect of selected probiotic strains (*Lactococcus lactis* subsp. *cremoris* YRC3780) on perceived stress and cortisol-related responses. The results showed reduced cortisol levels in saliva, as well as improved sleep quality and mental well-being among study participants [71]. A randomized study conducted by Jinxing Li et al. suggests that supplementation with *Bifidobacterium breve* 207-1 in healthy adult men exposed to high psychological stress, overweight, insomnia, and constipation affected the levels of neurotransmitters and HPA axis hormones via the microbiome–gut–brain axis [72]. Interestingly, the modulation of the HPA axis by probiotics does not necessarily translate into changes in cortisol levels themselves, as the latest clinical evidence indicates that certain probiotics formulations can alter the ACTH response without significantly affecting circulating cortisol levels [73].

Another important mechanism underlying the potential effects of psychobiotics is the modulation of the immune response, which may be particularly significant in the context of chronic neuroinflammation associated with neurodegenerative diseases. By influencing the composition and metabolic activity of microbiota, psychobiotics may reduce the host’s exposure to pro-inflammatory bacterial products, including LPS, and modulate cytokine production and the activity of immune cells. A reduction in the peripheral inflammatory response may, in turn, limit signals reaching the central nervous system and influence the activation of microglia, which is a key component of the brain’s neuroimmune response [56]. Peijun Tian and colleagues investigated the *Bifidobacterium breve* strain CCFM1025 in terms of its genomic and metabolic characteristics and psychobiotic potential, as well as its effects on gut microbiota and metabolic modulation. They demonstrated that administration of CCFM1025 to chronically stressed mice alter their gut microbiome and increases levels of xanthine, tryptophan, and short-chain fatty acids in the cecum, which in turn reduces depressive behaviors and neurological abnormalities [74].

Chronic microglial activation is one of the key components of the neuroinflammatory response observed in neurodegenerative diseases. Disruptions in the gut microbiota may contribute to the maintenance of a pro-inflammatory peripheral environment, whereas modulation of the microbiota by selected probiotic strains may reduce the severity of the inflammatory response and, consequently, influence microglial activation. Consequently, probiotics with presumed psychobiotic effects may potentially influence not only stress-related symptoms or behavioral disorders but also the pathophysiological processes underlying neurodegeneration [75]. Timothy R. Sampson et al. demonstrated that the gut microbiota was essential for the development of microglial activation, α-synuclein pathology, and motor deficits in mice with overexpression of α-synuclein. Depletion of the microbiota attenuated these pathological features, whereas recolonization with the microbiota restored the disease phenotype. Furthermore, administration of microbial metabolites, including short-chain fatty acids, promoted neuroinflammation and motor dysfunction, and microbiota transplantation from patients with Parkinson’s disease exacerbated motor impairments compared to microbiota from healthy donors [56]. In contrast, a study by Dong Y et al. using a mouse model of Parkinson’s disease showed that supplementation with *Bifidobacterium animalis* subsp. *lactis* NJ241 attenuated dopaminergic neuronal loss and glial activation while restoring gut microbial homeostasis and increasing intestinal SCFA levels. Importantly, the neuroprotective effects were associated with enhanced intestinal GLP-1 signaling and activation of PGC-1α in the substantia nigra, while pharmacological blockade of the GLP-1 receptor partially abolished the effects of the probiotic, suggesting the involvement of the gut-GLP-1-nigral PGC-1α pathway [76]. These apparently contrasting findings highlight that the effects of microbiota modulation are highly context- and strain-dependent and cannot be generalized across probiotic interventions. Similar observations have been reported in animal experimental models of Alzheimer’s disease [77,78]. Administration of *Lactiplantibacillus plantarum* HEAL9 to SAMP8 mice attenuated cognitive impairment and was associated with reduced microgliosis and astrogliosis, decreased accumulation of Aβ1-42, and suppression of NLRP3 inflammasome activation in both the gut and the brain. Normalization of circulating lipopolysaccharide-binding protein further suggested improved signaling related to the intestinal barrier, which supports the microbiota-gut-inflammasome-brain mechanism [78]. Available experimental data indicate that selected probiotic strains can influence many pathological processes associated with neurodegeneration, including intestinal barrier dysfunction, systemic inflammation, microglial activation, oxidative stress, protein homeostasis disorders, and neuronal survival. These effects appear to depend on the strain used and may be associated with the simultaneous modulation of several interrelated pathways within the microbiota–gut–brain axis. The processes mentioned above are not regulated exclusively by the gut microbiota. Trace elements also play a significant role in maintaining neuronal homeostasis, as they participate in numerous enzymatic and metabolic processes, including antioxidant defense, mitochondrial function, neurotransmission, and the regulation of the immune response. For this reason, disturbances in trace element homeostasis represent another potentially significant component of the pathogenesis of neurodegenerative diseases [79,80].

## 4. Trace Elements and Brain Health

### 4.1. Essential Trace Elements

The literature indicates that a total of 20 elements appear to be essential for the proper functioning of the human body. Half of these are metals, and the other half are nonmetals. Trace elements are micronutrients from the metal group that are essential for the proper functioning of the body [81]. The main trace elements include, among others, iron (Fe), zinc (Zn), copper (Cu), manganese (Mn), selenium (Se), molybdenum (Mo), and cobalt (Co) [82]. They are naturally supplied to the body through food and drinking water and are then absorbed by the gastrointestinal tract. Although they are present in the body in small amounts, they play a disproportionately large biological role. Fe, Zn, Cu, and Mn—referred to as “neurometals”—are of particular importance for the proper functioning of the brain [83]. They act as cofactors for many enzymes, participating in numerous enzymatic reactions within the cells of the central nervous system. Among other things, they influence neurotransmitter synthesis, myelination, neural information processing, and the protection of nerve cells. However, their balance within the body is crucial. A disruption in the homeostasis of these elements can lead to the formation of reactive oxygen species (ROS), neuronal damage, and, consequently, the development of neurodegenerative diseases [84].

### 4.2. Neuroprotective, Neurotoxic Effects and Neurodegeneration

**Figure 2:**
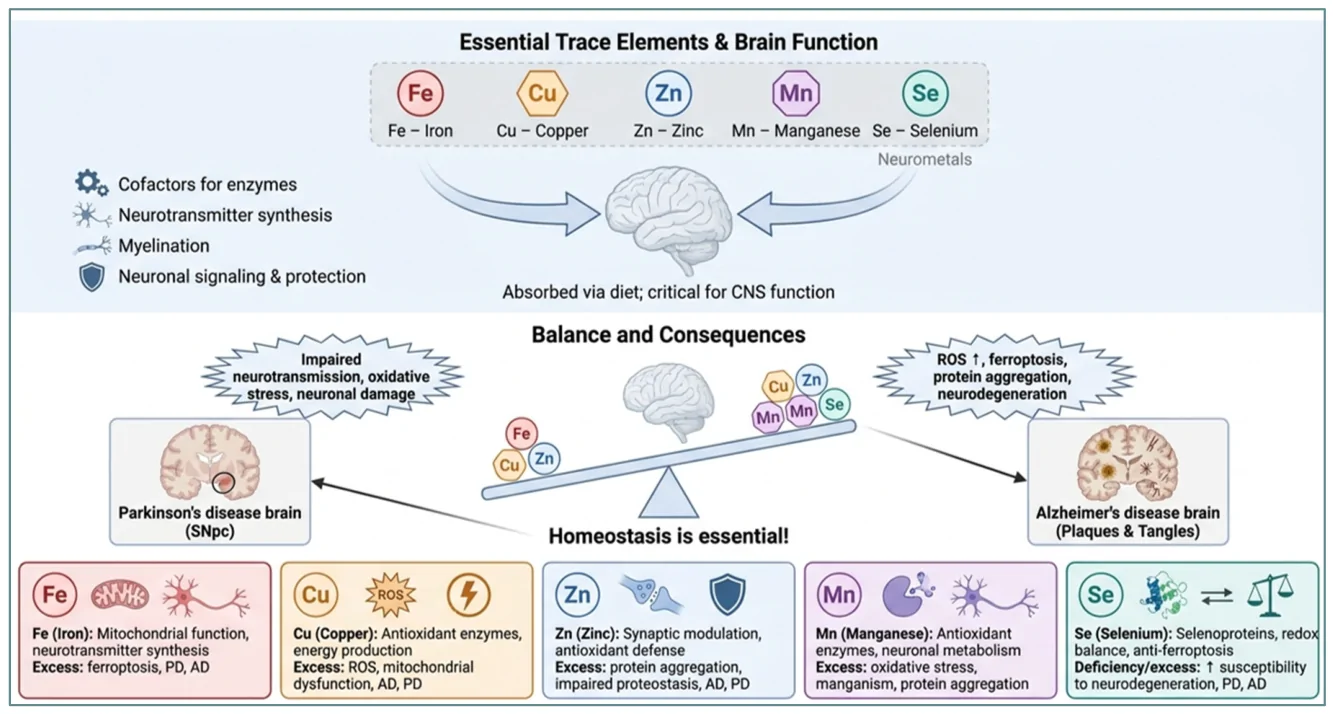
Essential Trace Elements and brain function.

Trace elements exhibit effects that depend on both their concentration and the biological context. Within physiological ranges, they are essential for the proper functioning of the nervous system and can perform important neuroprotective functions; however, their deficiency or excessive accumulation leads to a disruption of cellular homeostasis and may contribute to neurotoxicity (Figure 2) [83].

#### 4.2.1. Iron

Iron (Fe) is one of the key metals essential for the proper functioning of neurons. It participates in processes related to energy production and cellular metabolism. It is an important cofactor in the enzymatic synthesis of myelin, cholesterol, and fatty acids, which serve as building blocks for nerve cells [85]. As a component of iron-sulfur centers and heme groups, iron participates in the function of proteins in the mitochondrial electron transport chain, supporting the proper course of oxidative phosphorylation and ATP synthesis [86,87]. It is also a cofactor for tyrosine hydroxylase (TH) and tryptophan hydroxylase (TPH), enzymes involved in the biosynthesis of dopamine, norepinephrine, and serotonin, respectively—which are monoamine neurotransmitters involved in cognitive, emotional, and motivational processes [88,89].

Despite its significant physiological importance, iron can also exhibit neurotoxic effects under conditions of disrupted homeostasis. Of particular concern is its excessive accumulation in nervous tissue, which can lead to an increase in the pool of labile iron and an intensification of oxidative processes [90,91]. Disruptions in the homeostasis of this element are observed in neurodegenerative diseases. A specific mechanism of neurotoxicity associated with iron overload is ferroptosis. This is an iron-dependent form of regulated, non-apoptotic cell death characterized by the accumulation of redox-active iron, enhanced lipid peroxidation, and disruption of antioxidant systems [92,93]. Changes in iron homeostasis are particularly well documented in Parkinson’s disease (PD), in which elevated iron levels are observed in the substantia nigra, especially in its compact part (substantia nigra pars compacta, SNpc), associated with progressive dopaminergic degeneration and neuronal loss [94,95]. Neuroimaging studies have also demonstrated increased iron accumulation in the SNpc in patients with early-stage PD, and its quantification using quantitative magnetic susceptibility mapping (QSM) has enabled the differentiation between patients with PD and healthy individuals [96]. Abnormalities in iron distribution have also been described in Alzheimer’s disease. Postmortem brain tissue studies conducted by Junye Yao et al. demonstrated regional iron accumulation co-occurring with β-amyloid deposition, particularly in areas susceptible to neurodegenerative processes [97]. In turn, Mayr et al. observed strong ferritin expression in microglia and the presence of iron in microglia, droplet-like structures, and the cores of neuritic plaques, which is associated with the mechanism of ferroptosis and progressive degeneration [98]. Beyond iron, other trace-element disturbances are also relevant to neurodegeneration. Copper contributes to neuronal antioxidant defense and mitochondrial function [99], whereas postmortem analyses have revealed widespread selenium deficiency across the examined brain regions in Huntington’s disease [100].

#### 4.2.2. Copper

Copper (Cu) is a trace element classified as a neurometal. Cu is a cofactor for many enzymes, including the cytosolic Cu/Zn-SOD (SOD1) superoxide dismutase, which catalyzes the dismutation of the superoxide anion into hydrogen peroxide and oxygen, constituting an essential component of the cell’s enzymatic antioxidant defense [99]. It is essential for the proper functioning of the mitochondrial respiratory chain, as it serves as a cofactor for cytochrome c oxidase (COX, complex IV), the terminal enzyme of the electron transport chain [100,101].

A wide range of cellular processes in which copper plays regulatory and signaling roles—including those critical to the proper functioning of the CNS—occur only when copper levels remain balanced. Under conditions of deficiency resulting from diet or genetic factors, metabolic abnormalities, hypomyelination, and impaired neuronal arborization occur. Conversely, when copper is excessively available, its redox properties can lead to the uncontrolled generation of reactive oxygen species and an increase in oxidative stress. Excess Cu can disrupt mitochondrial function, increase lipid peroxidation, and lead to damage to proteins and cell membranes, ultimately contributing to neuronal dysfunction and death [102]. Interestingly, studies conducted on various types of human neurons indicate that mitochondria and the neuronal network are among the structures particularly susceptible to the toxic effects of excess Cu, and concurrent adverse changes in other elements, including Fe, Mn, Mg, and Ca, may further exacerbate its cellular consequences [103].

A growing body of evidence suggests that disturbances in copper homeostasis occur in the course of Alzheimer’s disease and Parkinson’s disease, although their nature and pathophysiological significance may vary among different neurodegenerative disorders [104]. Recent studies suggest that impaired Cu homeostasis may be directly linked to the pathological effects of amyloid-β (Aβ) on mitochondria. In AD models, an imbalance in the Cu(I)/Cu(II) ratio has been demonstrated, accompanied by an increase in mitochondrial Cu(I), elevated ROS production, and abnormalities in mitochondrial morphology and function [105]. Furthermore, other data points to the impact of Cu homeostasis disturbances on microglial function. In a mouse model it has been shown that excessive Cu accumulation in microglia was accompanied by a decrease in mitochondrial glutathione levels and an increase in oxidative stress. In the presence of Aβ, these phenomena were exacerbated and led to the release of oxidized mitochondrial DNA (ox-mtDNA), which activated the NLRP3 inflammasome and intensified the pro-inflammatory activation of microglia. At the same time, excess Cu disrupted cholesterol metabolism and limited the microglia’s ability to phagocytose Aβ, potentially promoting its accumulation [106]. In Parkinson’s disease (PD), impaired copper homeostasis is associated with reduced copper levels in the substantia nigra, which coexists with iron accumulation. A postmortem study demonstrated approximately 2.4-fold lower Cu levels in neuromelanin-containing neurons of the substantia nigra in patients with PD compared to controls, while Fe levels were increased approximately 4–5-fold [107]. In contrast, a study by Tae Young Kim et al. on cortical neurons were isolated from mice demonstrated that the pathogenic LRRK2 G2019S mutation (the most common genetic factor contributing to familial and sporadic PD) disrupts the redox state of the mitochondrial Cu chaperones, COX11, and SCO1, leading to abnormal metallation and assembly of cytochrome c oxidase and dysfunction of the mitochondrial electron transport chain. This suggests that in PD, not only the total Cu concentration but also its proper distribution and utilization by enzymes and mitochondrial chaperones may be significant [108].

#### 4.2.3. Zinc

Zinc (Zn) is another element essential for proper brain function. It is in the brain that Zn concentrations reach their highest level, at approximately 150 µmol/L [109]. It accumulates in the cerebral cortex, olfactory cortex, thalamus, hippocampus, and amygdala. In addition to acting as a cofactor for many enzymes, similar to the trace elements discussed above, Zn functions as a neuromodulator [110]. It is stored in the synaptic vesicles of glutamatergic neurons via the ZnT3 transporter and can be released into the synaptic cleft in a manner dependent on neuronal activity. Through this mechanism, it modulates the function of glutamatergic receptors and participates in the regulation of neuronal excitability, synaptic transmission, and neuronal plasticity [111]. Zinc’s neuroprotective effect is based on several mechanisms. It competes with iron and copper in the cell membrane, inhibiting the enzyme NADPH oxidase, thereby reducing chronic inflammation. Like copper, zinc is located in the active site of superoxide dismutase, which neutralizes ROS toxicity. Furthermore, it influences the expression of glutamate-cysteine ligase, which is responsible for limiting the rate of de novo glutathione synthesis [112]. Recent studies show that Zn may affect neuronal mitochondria. Bai et al. demonstrated that zinc inhibited the Lgals3-Bax pathway, reduced PANoptosis, limited mitochondrial dysfunction induced by oxidative stress, and thereby improved neuronal survival [113].

Despite Zn’s crucial role in maintaining neuronal homeostasis, its effects depend on concentration, cellular availability, and subcellular localization. Both deficiency and excessive accumulation can lead to impaired neuronal function. Of particular importance is an increase in the pool of labile, intracellular Zn, which under pathological conditions may contribute to impaired lysosomal function and autophagy. In a recent study by Hyun-Seung Lee et al. using a cellular model of Parkinson’s disease, it was demonstrated that inhibition of mitochondrial complex I led to an increase in mitochondrial reactive oxygen species and intracellular Zn concentration, which in turn promoted lysosomal membrane destabilization and neuronal death [114]. Disruptions in Zn homeostasis may also affect proteostasis. In Parkinson’s disease animal models, increased Zn availability has been associated with impaired ubiquitin-proteasome system function, mitochondrial dysfunction, and increased accumulation of α-synuclein [115]. Furthermore, biochemical studies indicate that Zn binding to α-synuclein may alter its conformation and accelerate the formation of amyloid structures [116]. This suggests that disturbances in zinc homeostasis may promote the accumulation of misfolded proteins, representing one of the potential mechanisms increasing the susceptibility of dopaminergic neurons to degeneration. The potential influence of zinc on the pathogenesis of Alzheimer’s disease has also been studied. An in vitro study by Li et al. demonstrated that the interaction of Zn with the R3 domain of the tau protein exacerbated its pathological changes, leading to increased levels of hyperphosphorylated tau, abnormalities in dendritic spine morphology, and autophagy dysfunction. Mechanistically, this phenomenon was accompanied by inhibition of the Akt/mTOR pathway and impaired fusion of autophagosomes with lysosomes, which may limit the proper removal of misfolded proteins [117].

#### 4.2.4. Manganese

Manganese (Mn) is another essential trace element involved in the regulation of numerous metabolic and cellular processes critical to the proper functioning of the nervous system. Adequate levels of manganese are necessary for the functioning of metabolic and antioxidant enzymes. Due to the presence of Mn in the active site of the mitochondrial superoxide dismutase SOD2, this element helps protect mitochondria from excessive accumulation of superoxide anion radicals and maintain redox balance [118]. Recent studies using neurons derived from induced pluripotent stem cells further indicate that proper Mn homeostasis is essential for mitochondrial bioenergetics, calcium homeostasis, and neuronal endocytosis [119].

Under conditions of excessive exposure to manganese and its accumulation, there is an increased risk of developing neurological damage [120]. One of the key mechanisms underlying this effect is the disruption of the redox balance, including increased production of reactive oxygen species, glutathione depletion, and impaired mitochondrial function [120]. A related, but distinct, mechanism has been demonstrated in experimental models of Parkinson’s disease, in which gut microbiota-derived TNF-α promoted dopaminergic neuron ferroptosis through the TNFR1–NF-κB–ATF4 signaling pathway [121]. This evidence links microbiota-associated inflammation with neuronal injury but should be distinguished from the mechanisms directly induced by manganese exposure. A growing body of evidence also points to the involvement of ferroptosis in neuronal damage caused by excessive Mn exposure. Experimental studies using in vivo (Mn-exposed mice) and in vitro (hippocampal HT22 cells) models have shown that excess Mn disrupts iron homeostasis, increases the redox-active iron pool, and enhances NCOA4-dependent ferritinophagy, leading to decreased levels of SLC7A11 and GPX4 and increased lipid peroxidation [122]. Chronic exposure to high concentrations of Mn can lead to manganism, a neurological syndrome characterized by symptoms of parkinsonism. Importantly, the mechanisms and neuropathological features of this disorder are not identical to those of idiopathic Parkinson’s disease [123]. Recent in vivo studies in mice indicate that Mn exposure leads to lysosomal dysfunction associated with a disruption of the Serpina3n-TFEB-v/p-ATPase pathway, which may impair α-synuclein degradation and contribute to damage to dopaminergic neurons [124]. Furthermore, in vitro studies suggest that Mn may directly promote α-synuclein aggregation by inducing changes in its structural organization [125]. These data suggest that disruption of manganese homeostasis may increase the dopaminergic system’s susceptibility to processes associated with protein aggregation, lysosomal dysfunction, and neuroinflammation. In the context of Alzheimer’s disease, available studies on mouse model and mouse-derived microglia and neuroblastoma cell lines indicate that excessive exposure to Mn may exacerbate β-amyloid pathology by activating microglia and increasing the pro-inflammatory response [126]. Furthermore, excess manganese may lead to cognitive impairments associated with ferroptosis, involving iron accumulation, lipid peroxidation, and impairment of antioxidant systems [127].

#### 4.2.5. Selenium

Selenium (Se) is a trace element essential for the proper functioning of the nervous system; its biological activity is primarily associated with its presence in selenoproteins in the form of selenocysteine. These proteins play a key role in maintaining redox homeostasis, protecting cells from oxidative damage, and preserving normal mitochondrial function [128]. Glutathione peroxidase 4 (GPX4) is considered particularly important in protecting neurons; it removes phospholipid peroxides and limits their accumulation, thereby serving as one of the most important mechanisms counteracting ferroptosis [129]. Selenoprotein P (SeP), on the other hand, plays a crucial role in selenium homeostasis within the central nervous system by participating in the transport and delivery of selenium to nerve cells [130]. A growing body of evidence suggests that the importance of selenoproteins extends beyond the direct regulation of oxidative processes. They may also contribute to maintaining the homeostasis of other elements essential for neuronal function. Among other things, it has been shown that selenoprotein K influences the palmitoylation of the transferrin receptor 1 (TfR1), thereby regulating iron homeostasis, mitochondrial function, and energy production in neurons [131]. In turn, the interaction between SeP and the apolipoprotein E2 receptor (ApoER2) enables the efficient delivery of selenium to cells and enhances their ability to resist ferroptosis [128]. As with other trace elements, the beneficial effects of selenium are strictly dependent on its availability and the maintenance of proper homeostasis; both selenium deficiency and excessive exposure can lead to an imbalance in the redox state.

The significance of disrupted selenium homeostasis is particularly evident in the context of neurodegenerative diseases, in which increased oxidative stress, mitochondrial dysfunction, iron metabolism disorders, and ferroptosis constitute major mechanisms of neuronal damage [128,132].

In their study using an in vitro model of Parkinson’s disease, Jung et al. demonstrated that selenium supplementation reduced ferroptosis in dopaminergic cells, likely through activation of the Nrf2/GPX4 axis. Increased selenium availability was associated with enhanced activity of antioxidant mechanisms and GPX4, which reduced lipid peroxidation and the death of dopaminergic cells. These results suggest that selenium may play a role in protecting dopaminergic neurons from ferroptosis-related damage; however, these observations are derived from experimental models and do not yet constitute proof of the efficacy of selenium supplementation in the prevention or treatment of Parkinson’s disease [133]. Similar observations were made in animal models of PD. In a model of 6-hydroxydopamine-induced neurotoxicity, the use of selenium nanoparticles reduced oxidative stress and lipid peroxidation and increased the activity of antioxidant enzymes, including glutathione peroxidase and superoxide dismutase. The observed improvement in the animals’ motor parameters suggests that increasing selenium bioavailability may limit damage to the dopaminergic system [134]. More recent strategies utilizing selenium nanoparticles also indicate the possibility of their targeted delivery to the CNS and the reduction of dopaminergic neuron death in MPTP models [135]. At the same time, data regarding the relationship between selenium concentration and PD in humans remain inconclusive. A meta-analysis found no significant difference in blood Se concentrations between PD patients and healthy individuals, whereas higher Se concentrations were observed in the CSF of PD patients [136]. In the case of Alzheimer’s disease, the potential significance of selenium is primarily linked to its role in maintaining redox homeostasis and controlling ferroptosis. The SELENOP-LRP8/ApoER2-GPX4 axis, responsible for delivering selenium to cells and maintaining the activity of key anti-ferroptotic mechanisms, may play a particular role [137]. The relationship between selenium, ferroptosis, and AD pathology has been investigated in preclinical studies. In the APP/PS1 model, the use of selenium nanoparticles targeted at the blood–brain barrier reduced Aβ42 aggregation, tau phosphorylation, glial cell activation, and mitochondrial dysfunction, accompanied by increased GPX1/4 activity and inhibition of neuronal ferroptosis. These effects were associated with improved cognitive function in the animals. The results suggest that disturbances in selenium metabolism may increase the susceptibility of neurons to neurodegenerative processes [138]. Overall, disturbances in the homeostasis of these five elements contribute to neuronal injury through overlapping but element-specific mechanisms. Excessive Fe, Cu, and Mn availability may promote ROS production, lipid peroxidation, and mitochondrial dysfunction, whereas Zn dyshomeostasis may impair mitochondrial and lysosomal function and contribute to α-synuclein and tau aggregation. In contrast, adequate Se availability supports antioxidant defense, particularly through GPX4-mediated inhibition of lipid peroxidation and ferroptosis. Collectively, these processes may converge on impaired energy metabolism, altered protein homeostasis, neuroinflammation, and ultimately reduced neuronal survival [90,91,92,93,94,95,96,97,98,102,103,104,105,106,107,108,113,114,115,116,117,118,119,120,121,122,123,124,125,126,127,128,129,130,131,132,133,134,135,136,137,138]. The main roles of the above-mentioned trace elements in physiological and pathological conditions are presented in Table 1.

## 5. Interactions Between Gut Microbiota, Trace Elements and Psychobiotics

### 5.1. Bidirectional Interactions Between Gut Microbiota and Trace Elements

Trace elements and the gut microbiota are in a dynamic, bidirectional relationship that may be important for maintaining homeostasis both within the gut and throughout the entire body. Elements such as iron, zinc, copper, and selenium—as discussed earlier—are essential not only for the proper functioning of host cells but also for the microorganisms inhabiting the gastrointestinal tract. They primarily function as cofactors or enzyme components involved in the basic metabolic processes of bacteria; consequently, their availability in the intestinal environment can influence microbial growth, biological activity, interspecies competition, and, thereby, the overall composition of the microbiome. At the same time, microorganisms can influence the local availability and bioavailability of elements, thereby potentially modifying their absorption and utilization by the host [139,140].

The effect of trace elements on the gut microbiota is complex and depends, among other factors, on their concentration, bioavailability, and the host’s baseline status. A particularly well-studied example is iron, which is essential for the functioning of numerous enzymes and metabolic pathways in microorganisms, while its availability in the intestinal lumen remains strictly regulated by the host. Microorganisms compete with the host for Fe, utilizing, among other things, siderophores and specialized transport systems with high affinity for this element [141]. At the same time, changes in Fe availability can modify the growth and competitive advantage of individual microorganisms. In a randomized study involving 923 infants, iron supplementation did not significantly alter the overall diversity or composition of the microbiota; however, exploratory analyses revealed a decrease in the abundance of *Bifidobacterium* and *Lactobacillus* and an increase in selected potentially pathogenic bacteria, with these changes being more pronounced in children with normal iron status before the start of the intervention [142]. In contrast, the most recent randomized trial in preterm infants showed that increasing the dose of enteral iron from 2 to 6 mg/kg/day improved iron status but did not lead to significant changes in the diversity or composition of the microbiota, indicating that the effect depends on the biological context and the characteristics of the study population [143]. A similar relationship was also observed for selenium and copper. In experimental studies, the effect of Se on microbiota composition varied depending on the dose administered and the host’s metabolic context, whereas for Cu, a clear relationship between the microbiota’s response and the concentration of this element was demonstrated [144,145,146].

This interdependence may also occur in the opposite direction. Microorganisms possess specialized mechanisms regulating intracellular metal homeostasis, including their uptake, binding, storage, and export. These mechanisms allow them to maintain the availability of metals essential for metabolic processes while simultaneously limiting their potential toxicity in the event of excessive exposure [147]. As a result, the relationship between the microbiota and trace elements can take the form of a feedback loop. A change in the availability of a given element may favor specific groups of microorganisms, while altered microbiota activity may in turn affect the bioavailability and absorption of that element by the host. This mechanism has been particularly well documented regarding iron. It has been shown that metabolites produced by gut bacteria can modulate HIF-2α and ferritin expression, thereby influencing intestinal Fe transport and its systemic homeostasis [148]. Recent experimental data further indicate that modulating the microbiota with *Bifidobacterium animalis* subsp. *lactis* Ca360 may be associated with improved intestinal iron metabolism, changes in microbiota composition, and improvements in iron deficiency parameters. Thus, the interaction between element availability and microbiota activity may create a dynamic feedback loop whose consequences may extend beyond the intestinal environment and potentially influence systemic trace element homeostasis [149].

### 5.2. Interplay Between Psychobiotics, Trace Elements, and Intestinal Homeostasis

The bidirectional relationship between the gut microbiota and trace elements raises the question of whether targeted modulation of the gut microbial ecosystem can also influence the homeostasis of micronutrients. In this context, psychobiotics may play a particularly important role, as their biological activity extends beyond influencing the composition of the microbiota itself and may encompass broader changes in metabolic and functional processes occurring within the gut. Although direct evidence linking psychobiotic interventions to trace element homeostasis remains limited, studies of conventional probiotics may provide complementary mechanistic insights into microbiota–trace element interactions. In this section, such studies are considered indirect evidence when they assess mineral homeostasis or intestinal function without investigating psychological, cognitive, or neurodegenerative outcomes. Importantly, the observed effects appear to depend on the specific strain used, the chemical form and dose of the given element, as well as the host’s baseline nutritional and metabolic status. One well-studied example is the interaction between *Lactiplantibacillus plantarum* 299v and iron. This strain has also been investigated for its psychobiotic properties in a randomized, placebo-controlled clinical trial involving patients with major depressive disorder, in which adjunctive supplementation during SSRI treatment improved selected cognitive measures and reduced plasma kynurenine concentrations [150]. However, its psychobiotic effects and its association with iron status have been investigated in separate clinical studies. In a randomized clinical study involving 295 patients with iron-deficiency anemia, oral iron replacement providing 100 mg elemental iron/day was combined with *L. plantarum* 299v supplementation (10^10^ CFU/day) during the first 30 days of treatment. Over the three-month follow-up, probiotic supplementation was associated with improved gastrointestinal tolerance and more favorable changes in selected iron-status parameters compared with iron supplementation alone [151]. As this study did not assess psychological or cognitive outcomes, it provides indirect evidence of an interaction between a strain with independently documented psychobiotic properties and iron supplementation, rather than direct evidence of a psychobiotic–iron mechanism.

This suggests that modulation of the gut microbiome may influence the body’s biological response to trace element supplementation. Data from experimental models further indicate that probiotic microorganisms can affect trace element homeostasis under conditions of both deficiency and excess. In a mouse model study, administration of *Lacticaseibacillus casei* (0.1 mL at 2 × 10^9^ CFU/mL) Zhang to animals exposed to a high-iron diet alleviated a number of changes associated with iron overload, including disturbances in iron homeostasis parameters, IL-22 signaling, gut microbiota composition, and its metabolic functions [152]. Importantly, the beneficial effect of this strain on iron homeostasis parameters was less pronounced in animals lacking IL-22, suggesting that host immune signaling may serve as an intermediate mechanism linking probiotic activity to iron homeostasis. In contrast, in a study using a zinc-deficient mouse model, the concurrent administration of *Bifidobacterium animalis* subsp. *lactis* Ca360 (10^9^ CFU/mL) and zinc sulfate (6.96 mg Zn/kg body weight/day) improved zinc status, increased the activity of antioxidant enzymes, reduced oxidative stress and inflammation in the intestine, and alleviated mucosal damage. The intervention was also accompanied by changes in the gut microbiota profile and increased expression of genes involved in intestinal zinc transport and storage [153]. The results obtained indicate a potential interdependence between microbiota modulation and the availability of the element. A 2026 randomized clinical trial involving 84 patients with irritable bowel syndrome (IBS) evaluated daily supplementation with 20 mg elemental zinc alone or combined with a multistrain probiotic preparation (10^9^ CFU) for 90 days. Both interventions increased serum zinc concentrations and improved IBS symptom severity compared with placebo, whereas zinc alone was more effective in reducing depression and stress scores, indicating that combined supplementation does not necessarily produce additive psychobiotic effects [154]. Taken together, these findings suggest that probiotic-associated modulation of trace-element homeostasis may involve several pathways, including changes in the expression of intestinal mineral transport and storage-related genes [153] and host immune signaling, as indicated by the IL-22-dependent effects observed in iron-overloaded mice [152]. In addition, microbial metabolites such as 1,3-diaminopropane and reuterin have been shown to regulate HIF-2α activity and ferritin expression, thereby influencing intestinal iron absorption and storage [148]. However, this latter mechanism represents evidence of microbiota-derived metabolite signaling rather than a directly demonstrated mechanism of the specific probiotic strains discussed above. Furthermore, although clinical studies have reported improvements in selected iron and zinc status parameters following probiotic co-supplementation [150,154], the contribution of microbiota-mediated changes in mineral absorption has not been directly established.

**Figure 3:**
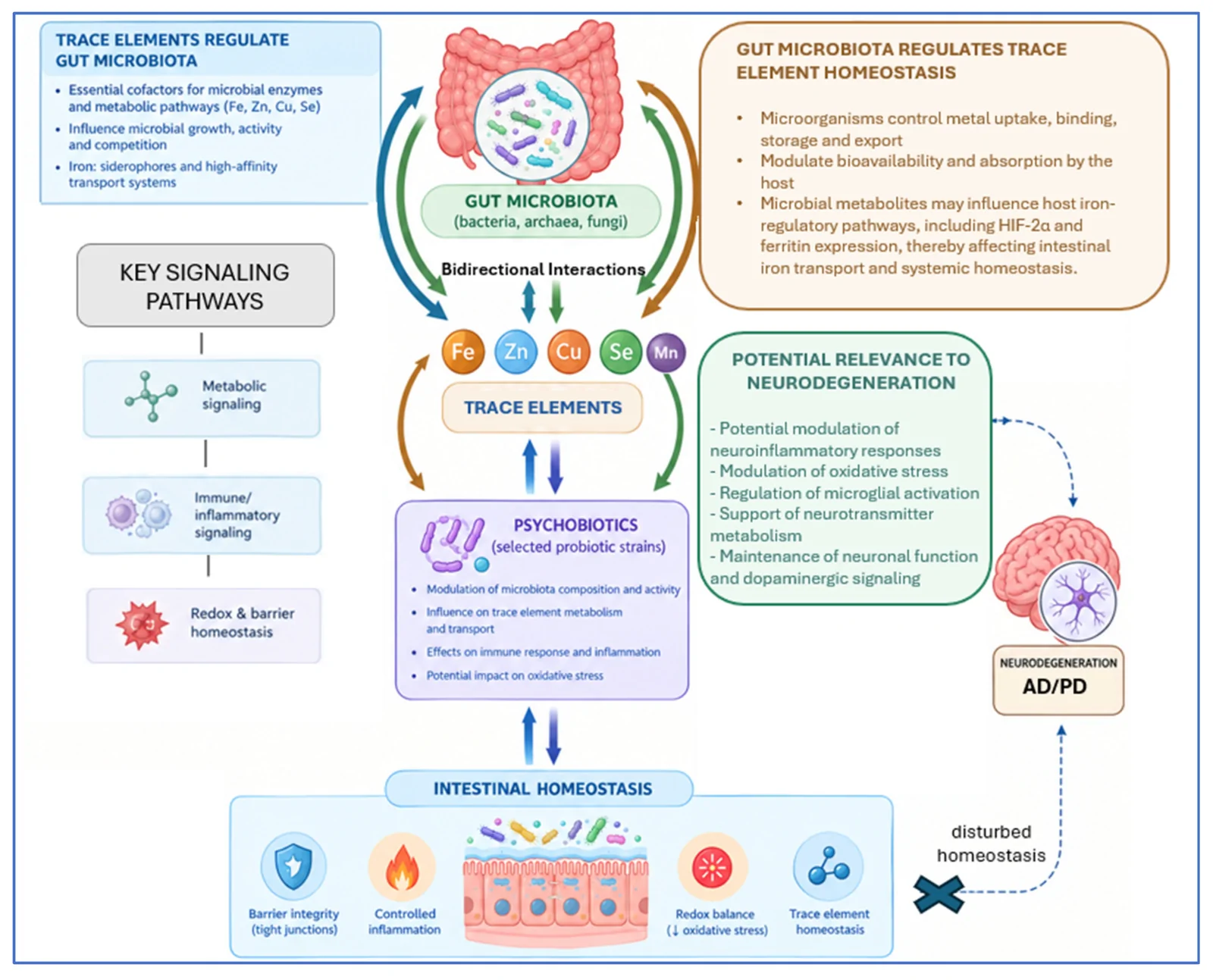
The potential role of gut microbiota, trace elements and psychobiotics in neurodegeneration.

The direction of this relationship can also be reversed, where the availability of a trace element influences the biological properties of probiotic microorganisms. An example of this is selenium, which was a key element in an experimental study. The study analyzed lactic acid bacteria enriched with this element (supplied as selenomethionine, or inorganic selenium, supplied as sodium selenite, at concentrations of 5 or 250 μM), demonstrating that the incorporation of Se into *Lactiplantibacillus plantarum* can modify the bacteria’s antioxidant properties and the way selenium is utilized, with the effect depending on its chemical form and the concentration used [155]. Selenium-enriched bacterial preparations also influenced the antioxidant response of intestinal epithelial cells, suggesting that enriching probiotic microorganisms with trace elements may modify not only the nutritional value of such an intervention but also its biological activity in the intestinal environment. A similar approach was applied to preparations containing *Limosilactobacillus reuteri* and selenium compounds, in which the use of a selenium-based formulation improved bacterial survival in the gastrointestinal tract and enhanced the strain’s beneficial effects on oxidative stress, inflammation, and microbiota homeostasis in an experimental model of colitis [156]. In an experimental study in male Swiss mice, a selenium-enriched *Lactobacillus reuteri* LRE02 postbiotic was administered intragastrically for 14 days at a dose containing 60 µg selenium per animal. The selenium-enriched preparation exhibited antidepressant- and anxiolytic-like effects and modulated intestinal redox and PI3K/Akt/mTOR signaling pathways, providing preclinical evidence that selenium enrichment may influence the psychobiological activity of microbial preparations [157]. An experimental study in C57BL/6 mice demonstrated that dietary supplementation with selenium-enriched *Pichia kudriavzevii* altered gut microbiota composition and host metabolism in a dose-dependent manner. The intervention enhanced antioxidant capacity, reduced inflammatory responses, stimulated short-chain fatty acid production, and affected tryptophan- and tyrosine-related metabolic pathways, suggesting a functional link between selenium-enriched microorganisms, intestinal homeostasis, and microbial metabolic activity [144]. These findings suggest that psychobiotics and trace elements should not be viewed as two independent factors affecting the host, but rather as components of an interdependent biological environment (Figure 3).

### 5.3. From the Gut to the Brain: Implications for Neurodegeneration

Interactions between the gut microbiota and trace elements, as well as their potential modulation by psychobiotics, may have consequences not only within the local gut environment but also extend far beyond it. The availability and metabolism of trace elements can influence the functional activity of microorganisms, while changes in the microbiota can in turn modify the bioavailability and homeostasis of these components in the body. The simultaneous use of psychobiotic interventions can further modulate and reshape the environment in which these processes occur. As a result, the gut may serve as a hub for the integration of metabolic, immunological, and micronutrient-related signals, the effects of which can be transmitted to distant tissues, including the central nervous system. One example illustrating the importance of such a link is research on manganese. In a mouse model, chronic exposure to Mn led to changes in the composition of the gut microbiota and an exacerbation of pathological changes in the brain, including increased expression of Aβ, tau, and phosphorylated tau. Importantly, microbiota transplantation from healthy animals partially reversed the changes associated with Mn exposure and attenuated neurotoxicity, accompanied by reduced activation of the cGAS-STING/NLRP3 pathway [158]. This suggests that the microbiota may not only co-occur with changes induced by disturbances in elemental homeostasis but also participate in modulating their consequences for the nervous system. In turn, Haitao Dong et al., using 16S rRNA analysis and metabolomics, demonstrated that chronic Mn exposure in mice induced dysbiosis and was accompanied by metabolomic changes in the striatum [159]. A particularly evident link between microorganisms, trace elements, and brain function was observed in a study by Lei Qiao et al. using a model of cognitive impairment, in which *Lactobacillus casei* ATCC 393 enriched with selenium nanoparticles was administered. The intervention using Se-enriched *L. casei* was associated with improved cognitive function, beneficial changes in microbiota composition, and improved parameters related to intestinal barrier integrity and the oxidative-antioxidant balance. At the same time, a reduction in neuropathological changes was observed, including Aβ aggregation, tau hyperphosphorylation, and microglial activation [160]. Clinical data also provide preliminary evidence that modulation of the microbiota may be associated with changes in cognitive function. In a randomized, double-blind study involving 100 individuals with mild cognitive impairment, 12-week supplementation with *Lactobacillus delbrueckii* subsp. *lactis* CKDB001 led to greater improvement in ADAS-Cog13 scores and selected parameters of memory and executive function compared to placebo. At the same time, analysis of the gut microbiota and fecal metabolome revealed changes in the composition of microorganisms and the metabolite profile, particularly within the families *Lactobacillaceae* and *Bifidobacteriaceae* and the genus *Lactobacillus* [161]. These results provide a clinical example of the co-occurrence of changes in the gut environment and cognitive function, supporting the concept of microbiota’s role in gut–brain communication. Similar observations were made in studies of patients with Parkinson’s disease. In a multicenter, randomized trial, supplementation with four probiotic strains led to changes in the composition of the microbiota and a reduction in TNF-α levels and was associated with improvements in selected clinical parameters. Importantly, no changes in SCFA concentrations were observed, suggesting that the effects of microbiota modulation may also involve mechanisms beyond changes in these metabolites. In the context of the data presented earlier, this suggests that the microbiota may serve as a functional link between the intestinal environment and processes critical to nervous system function and the development of neurodegeneration [162]. In a 12-week randomized, double-blind, controlled trial involving 79 patients, co-supplementation with selenium (200 μg/day) and *Lactobacillus acidophilus*, *Bifidobacterium bifidum*, and *Bifidobacterium longum* significantly improved MMSE scores compared with selenium alone and placebo. The combined intervention also reduced hs-CRP and improved total antioxidant capacity, glutathione levels, and selected indices of insulin resistance [163]. In another randomized, placebo-controlled trial involving 90 patients with mild to moderate AD, 12-week supplementation with *Bifidobacterium longum* R0175 or *Lactobacillus rhamnosus* HA-114 improved cognitive performance, with additional benefits observed for verbal fluency, instrumental activities of daily living, and anxiety, although the effects varied across outcomes [164]. In PD, a randomized controlled trial demonstrated that a fermented milk product containing probiotics and prebiotic fiber increased the frequency of complete bowel movements compared with placebo, supporting the clinical relevance of targeting gastrointestinal dysfunction in this population [165]. Further evidence linking trace element homeostasis to the gut–brain axis comes from a recent study reporting lower hair iron and copper and higher manganese and arsenic levels in patients with PD. Complementary experiments in an MPTP-induced mouse model associated intestinal barrier impairment and altered host and microbial iron-handling pathways with systemic iron deficiency [166]. The available evidence suggests partly disease-specific patterns of interaction between psychobiotics and trace elements. In AD, these mechanisms are particularly relevant to Aβ accumulation, tau pathology, and neuroinflammation, as illustrated by experimental studies investigating probiotic interventions, including selenium-enriched *L. casei* [78], as well as the effects of trace-element dyshomeostasis. In PD, probiotic studies have primarily focused on dopaminergic neuronal integrity and neuroinflammatory responses [76], whereas trace-element-related mechanisms have also been associated with α-synuclein pathology and mitochondrial dysfunction. However, although promising findings have been reported in experimental models, direct evidence of probiotic-mediated modulation of α-synuclein aggregation and of combined psychobiotic–trace element effects remain limited, particularly in PD and in human studies. Experimental and clinical evidence linking gut microbiota, trace elements, and neurological outcomes in neurodegenerative diseases is summarized in Table 2.

## 6. Limitations and Future Perspectives

Despite a growing body of evidence pointing to the importance of the gut microbiota, psychobiotics, and trace elements in neurodegenerative processes, direct evidence regarding their interactions remains limited. Most studies to date have analyzed individual components of this system independently, and a significant portion of the available data comes from in vitro studies and animal models. Clinical trials remain relatively few in number and are characterized by considerable methodological heterogeneity, which limits the ability to directly compare results. Moreover, current clinical studies provide limited information on long-term safety, potential adverse effects, and interactions between probiotic preparations and trace-element supplementation. Optimal dose ranges and treatment durations, particularly for combined interventions, remain to be established, as responses may vary according to probiotic strain, chemical form and bioavailability of the element, and baseline nutritional status. Furthermore, differences in microbiota composition and the characteristics of the study populations may further influence the results obtained. Another challenge remains translating experimental results to clinical settings. Differences between animal models and humans in terms of microbiota composition, metabolism, immune response, and trace element homeostasis may limit the translation of preclinical observations. Given that neurodegenerative diseases are characterized by complex etiopathology and a long-term course, this makes it difficult to replicate them in short-term experimental models.

Future research should focus on designing long-term clinical studies that integrate microbiota profiling and composition at the strain level, assessment of trace element status, and changes in these parameters during psychobiotic interventions. Such an approach could help answer whether the observed associations are causal and whether individual differences in the microbiota and trace element homeostasis influence the response to psychobiotics. In the long term, this may help to better understand the significance of the microbiota-trace elements-psychobiotics axis in the pathogenesis and progression of neurodegenerative diseases and lay the groundwork for developing more precise strategies to support the nervous system.

## 7. Conclusions

Available data indicates that the gut microbiota, psychobiotics, and trace elements form an interconnected system that may participate in the regulation of processes critical to nervous system function and the development of neurodegeneration. The interactions between these elements may represent an important, yet insufficiently understood, component of the microbiota–gut–brain axis. This review brings together two research areas that have been studied separately: psychobiotic interventions and trace-element homeostasis. It explores how trace elements may influence the composition and activity of the gut microbiota, while microbial processes may, in turn, affect trace-element availability and host homeostasis. Although current evidence suggests the potential importance of this system in neurodegeneration, the lack of sufficient clinical data does not yet allow for a determination of its therapeutic significance. Therefore, further research into these relationships may contribute to a better understanding of the mechanisms of neurodegeneration and the personalization of new therapeutic strategies.

## Figures and Tables

**Table 1:** The role of trace elements in health and neurodegeneration.

Element	Physiological Role	Pathological Imbalance	AD/PD Relevance	Ref.
Iron (Fe)	Supports mitochondrial electron transport, oxidative phosphorylation and ATP synthesis; serves as a cofactor for enzymes involved in myelin, cholesterol, fatty acid and monoamine neurotransmitter synthesis.	Excess accumulation increases the labile iron pool, promotes oxidative processes and lipid peroxidation, and may trigger ferroptosis.	PD: Iron accumulation in the substantia nigra pars compacta is associated with dopaminergic degeneration; increased iron can be detected in early-stage PD. AD: Regional iron accumulation co-occurs with β-amyloid deposition; iron-containing microglia and neuritic plaques may be associated with ferroptosis and neurodegeneration.	[85,86,87,88,89,90,91,92,93,94,95,96,97,98]
Copper (Cu)	Cofactor for SOD1, contributing to antioxidant defense, and for cytochrome c oxidase (complex IV), supporting mitochondrial function.	Deficiency may cause metabolic abnormalities, hypomyelination and impaired neuronal arborization. Excess may increase oxidative stress, disrupt mitochondrial function, enhance lipid peroxidation and damage cellular structures.	AD: Altered Cu homeostasis is associated with Aβ-related mitochondrial dysfunction, oxidative stress, microglial NLRP3 inflammasome activation and impaired Aβ phagocytosis. PD: Reduced Cu levels in the substantia nigra coexist with Fe accumulation; altered Cu handling may impair cytochrome c oxidase assembly and mitochondrial function.	[99,100,101,102,103,104,105,106,107,108]
Zinc (Zn)	Acts as an enzymatic cofactor and neuromodulator; regulates glutamatergic neurotransmission, neuronal excitability and plasticity. Contributes to antioxidant defense and glutathione synthesis and may protect neuronal mitochondria.	Both deficiency and excess can impair neuronal function. Increased labile Zn may destabilize lysosomal membranes, impair autophagy and proteostasis, disrupt mitochondrial function and promote neuronal death.	PD: Increased Zn availability is associated with impaired ubiquitin–proteasome function, mitochondrial dysfunction and α-synuclein accumulation and aggregation. AD: Zn interaction with tau may increase tau hyperphosphorylation, alter dendritic spine morphology and impair autophagy.	[109,110,111,112,113,114,115,116,117]
Manganese (Mn)	Supports metabolic and antioxidant enzymes, particularly mitochondrial SOD2, and contributes to redox balance, mitochondrial bioenergetics, calcium homeostasis and neuronal endocytosis.	Excess exposure promotes ROS production, glutathione depletion, mitochondrial dysfunction, iron dysregulation and ferroptosis; it may also impair lysosomal function and protein degradation.	PD: Chronic excess can cause manganism, with parkinsonian symptoms; Mn may promote α-synuclein aggregation and impair its lysosomal degradation. AD: Excess Mn may exacerbate β-amyloid pathology through microglial activation and inflammation and contribute to cognitive impairment through ferroptosis.	[118,119,120,121,122,123,124,125,126,127]
Selenium (Se)	Supports selenoprotein-mediated redox homeostasis, mitochondrial function and protection against ferroptosis. GPX4 removes phospholipid peroxides, while SeP contributes to selenium delivery in the CNS. Selenoprotein K also regulates iron homeostasis.	Both deficiency and excessive exposure may disturb redox balance and compromise neuronal homeostasis. Impaired selenium-dependent antioxidant mechanisms may increase susceptibility to ferroptosis.	PD: Experimental models suggest that selenium can reduce ferroptosis and oxidative damage in dopaminergic neurons; human blood concentration findings remain inconclusive. AD: The SELENOP–LRP8/ApoER2–GPX4 axis may protect against ferroptosis; selenium nanoparticles reduced Aβ aggregation, tau phosphorylation, glial activation and neuronal damage in an animal model.	[128,129,130,131,132,133,134,135,136,137,138]

**Table 2:** Experimental and Clinical Evidence Linking Gut Microbiota, Trace Elements, and Brain Function.

Study Model/Neurological Outcome	Microbiota-Related Findings	Trace Element/Intervention	Brain-Related Findings and Evidence Linking the Gut to the Brain	Ref.
Mouse model of manganese-induced neurotoxicity.	Chronic manganese exposure altered gut microbiota. Fecal microbiota transplantation (FMT) from healthy mice partially reversed the microbiota-associated changes.	Manganese chloride exposure (Mn content 200 mg/L in drinking water); FMT was the microbiota-targeted intervention.	Mn exposure was associated with changes in brain amyloid-β (Aβ), tau, and phosphorylated tau. FMT attenuated neurotoxicity and reduced cGAS–STING/NLRP3 pathway activation, suggesting that the gut microbiota may modify the neurological effects of Mn exposure.	[158]
Mouse model of manganese-induced neurotoxicity.	Chronic Mn exposure induced gut dysbiosis.	Chronic intranasal MnCl_2_ exposure; serum Mn increased. The exact administered concentration is not stated in the available abstract.	Gut dysbiosis was accompanied by metabolomic alterations in the striatum, indicating an association between intestinal microbial changes and brain metabolism.	[159]
Experimental model of cognitive impairment.	Selenium-enriched *Lactobacillus casei* ATCC 393 improved gut microbiota composition and intestinal barrier integrity.	The Se-enriched diet contained 0.3 mg Se/kg and 0.5 × 10^9^ CFU/g of *L. casei* ATCC 393-SeNPs; the Se-deficient diet contained <0.015 mg Se/kg. SeNPs were prepared using 1.2 mM sodium selenite during bacterial synthesis.	The intervention improved cognitive performance, reduced Aβ aggregation, tau hyperphosphorylation, and microglial activation, and improved oxidative-antioxidant balance. These findings suggest potential links between selenium-based probiotic intervention, intestinal homeostasis, and neuroprotection.	[160]
Clinical study in patients with mild cognitive impairment (MCI); randomized, double-blind trial.	Probiotic supplementation altered the gut microbiota and fecal metabolome, including changes in *Lactobacillaceae*, *Bifidobacteriaceae*, and *Lactobacillus*.	*Lactobacillus delbrueckii* subsp. *lactis* CKDB001; no trace-element outcomes reported.	Improvements in ADAS-Cog13 scores and selected memory and executive-function measures occurred alongside microbiota and fecal metabolome changes. The study supports a potential association between probiotic-induced intestinal changes and cognitive outcomes but does not directly establish a role for trace elements.	[161]
Clinical study in patients with Parkinson’s disease (PD); multicenter randomized trial.	Supplementation with a four-strain probiotic altered gut microbiota composition.	Four-strain probiotic intervention; no trace-element outcomes reported.	The intervention reduced TNF-α and improved selected clinical parameters, whereas short-chain fatty acid (SCFA) concentrations remained unchanged. The findings suggest potential microbiota-associated clinical effects but do not directly demonstrate microbiota–trace-element interactions.	[162]
Clinical study in patients with Alzheimer’s disease (AD); randomized, double-blind, controlled trial; 79 participants.	Probiotic supplementation was administered, but direct changes in gut microbiota composition were not reported as a principal outcome in the study.	Selenium (200 µg/day) alone or combined with a probiotic mixture containing *Lactobacillus acidophilus*, *Bifidobacterium bifidum*, and *B. longum* (2 × 10^9^ CFU/day of each strain).	Combined probiotic and selenium supplementation improved MMSE scores and selected inflammatory and antioxidant parameters compared with selenium alone or placebo. This study provides clinical evidence for a combined probiotic–trace-element intervention, although direct microbiota changes were not established.	[163]
Clinical study in patients with mild-to-moderate AD; randomized, double-blind, placebo-controlled trial; 90 participants.	Participants received single-strain probiotics; gut microbiota composition was not directly assessed as a principal outcome.	*Lactobacillus rhamnosus* HA-114 or *Bifidobacterium longum* R0175; no trace-element outcomes reported.	Twelve weeks of probiotic supplementation was associated with improved cognitive performance measured by the MMSE, particularly in the *B. longum* group. The findings support clinical investigation of probiotics in AD but do not establish a direct microbiota–trace-element link.	[164]
Clinical study in patients with PD and constipation; randomized, double-blind, placebo-controlled trial; 120 participants.	A fermented milk containing multiple probiotic strains and prebiotic fiber was administered. Direct microbiota composition changes were not the primary outcome.	Probiotic and prebiotic intervention; no trace-element outcomes reported.	The intervention increased the number of complete bowel movements per week compared with placebo. The study supports the clinical relevance of microbiota-targeted interventions for gastrointestinal symptoms in PD but does not directly demonstrate effects on neurodegeneration or trace-element homeostasis.	[165]
Clinical case–control study in patients with PD; 60 patients and matched controls, with complementary experiments in a mouse model.	Gut barrier dysfunction and altered microbial iron-acquisition pathways were investigated in the experimental component; these mechanisms were not directly demonstrated in the human cohort.	Hair metal profile: lower iron and copper, higher manganese and arsenic, and unchanged zinc in patients with PD.	The human data identified altered hair metal profiles in PD. Complementary mouse experiments linked intestinal barrier dysfunction and microbial iron-acquisition pathways to altered iron homeostasis, providing a potential mechanistic connection between the gut and systemic metal status.	[166]
Randomized, double-blind clinical trial in 84 patients with irritable bowel syndrome (IBS); 90 days.	A multistrain probiotic (10^9^ CFU/day) was given with zinc in one intervention arm; microbiota composition was not reported as a principal outcome.	Zinc gluconate providing 20 mg elemental Zn/day, alone or combined with a nine-strain probiotic preparation; serum Zn and TNF-α were assessed.	Zinc with or without probiotics improved serum Zn and IBS symptom severity. Zinc alone produced more favorable depression/stress outcomes than the combined intervention; these are mood-related findings, not evidence in a neurodegenerative-disease model.	[154]
Experimental study in male Swiss mice (*n* = 7/group); intragastric treatment once daily for 14 days.	Live *Limosilactobacillus reuteri* LRE02, inactivated postbiotic, and selenium-enriched inactivated postbiotic were compared; intestinal redox, inflammatory and signaling markers were assessed.	300 μL per administration at 10^9^ CFU/mL; selenium-enriched postbiotic contained 60 μg Se per dose and was prepared using sodium selenite. Host Se status was not reported as an outcome.	All *L. reuteri*-based preparations showed antidepressant-like effects; the Se-enriched postbiotic also showed anxiolytic-like effects and modulated redox/signaling markers. This is preclinical behavioral evidence, not evidence of improved systemic Se homeostasis.	[157]
Experimental dietary-supplementation study in mice; low- and high-selenium-enriched yeast preparations were compared.	Selenium-enriched *Pichia kudriavzevii* (yeast) altered gut microbiota and stimulated SCFA production; changes in tryptophan-, tyrosine- and other metabolic pathways were reported.	Dietary Se-enriched *P. kudriavzevii*; low- and high-dose preparations were compared.Host Se status was not identified as a direct endpoint in the abstract.	No brain, behavioral or cognitive outcomes were assessed. The findings provide mechanistic evidence linking selenium-enriched microbial preparations to gut ecology and host metabolism, rather than direct evidence of neuroprotection.	[158]

## Data Availability

No new data were created or analyzed in this study. Data sharing is not applicable to this article.
